# 

**Published:** 1886-05-20

**Authors:** 


### TABLE OF CONTENTS.

ORIGINAL AND SELECTED ARTICLES.

On the Use of the Muriate of Cocaine in Diseases of the Throat, by P. R. Cortelyou, M.D., Marietta, Ga. ... 165
The Administration of Anæsthetics, by Jos. W. Hearn, M.D. ... 167
The Braxton Hicks Method of Treating Placenta Prævia ... 173
Fœtal Psychology, by Thos. S. Sozinskey, M.D., PH.D., of Philadelphia ... 175
Therapeutic Notes, by Dr. J. J. Berry, of Portsmouth, N. H. ... 176
Malignant Pustule, by Dr. F. W. Moffitt, Randolph, Wis ... 178
Diphtheria — Croup — O'Dwyer's Method — Recovery ... 179

ABSTRACTS AND GLEANINGS.

Retention of Urine; Paraldehyde ... 181
Amenorrhœa ... 182
Veratrum in Typhoid Fever ... 183
Arsenic in Lymphadenoma; The treatment of Painful Fissure of Anus Without Operation ... 184
Tobacco; Cocaine in Pruritus Ani ... 185
A Remedy for Hay Fever; The Arsenic Habit ... 186
Salicylic Lemonade; Rapid Cure of Suppuration of Middle Ear of 35 Years' Standing; Pilocarpin Injections ... 187
A Portugese Method of Treating Ringworm; Treatment of Angina Pectoris; Benzoate of Cocaine ... 188
The Digestion of Milk; in Coryza; Injections of Oil of Turpentine for the Radical Cure of Fistulæ ... 189
Chloride of Calcium in Glandular Enlargements; Atropia Hypodermically to Prevent the Heart Depressant Effects of Chloroform; Lactation and Medicaments ... 190
Corning on Local Anæsthesia; Suicides ... 191
Cimicifuga for Headache; To Arrest Nasal Hemorrhage ... 192
How to Treat Bubo; Artificial Feeding of Infants; Cocaine in Tonsillotomy ... 193

SCIENTIFIC ITEMS.

Effect of 280,000 Pounds of Explosives 190 Miles Away; Experimental Yellow Fever; How Far Light Penetrates Deep Sea Depths ... 194
Guthrie's Telephone; Walnut Hair Dye ... 195

PRACTICAL NOTES AND FORMULÆ.

Pruritus Vulvæ; For Rheumatism; Elixir of Chloroform; For the Typhoid Condition ... 196

EDITORIALS AND MISCELLANEOUS.

Editorial Notices; New Advertisements ... 197
Medical Association of Georgia ... 198
Publication of the Transactions of the Medical Association of Georgia ... 201
In Memoriam; Medical Association of Ala.; Books and Pamphlets Received ... 203
Receipted; Special Notices ... 204

